# Nursing students’ experiences from clinical education using the TPSN model

**DOI:** 10.1186/s12912-024-01810-6

**Published:** 2024-03-04

**Authors:** Vahid Zamanzadeh, Leila Valizadeh, Akram Ghahramanian, Maryam Namadi-Vosoughi, Farzaneh Bagheriyeh, Afsaneh Pourmollamirza

**Affiliations:** 1 Department of Medical-Surgical Nursing, School of Nursing and Midwifery, Shahid Beheshti University of Medical Sciences, Tehran, Iran; 2 Department of Pediatric Nursing, School of Nursing and Midwifery, Shahid Beheshti University of Medical Sciences, Tehran, Iran; 3 Department of Medical-Surgical Nursing, School of Nursing and Midwifery, Tabriz University of Medical Sciences, Tabriz, Iran; 4 Department of Medical-Surgical Nursing, School of Nursing and Midwifery, Urmia University of Medical Sciences, Urmia, Iran; 5 Department of Geriatric Nursing, School of Nursing and Midwifery, Shahid Beheshti University of Medical Sciences, Shahid Chamran Highway, 1996835119 Tehran, Iran

**Keywords:** Nursing, Nursing student, Theory-practice gap, Clinical education, TPSN model

## Abstract

**Background:**

The TPSN model is an innovative model to create an integration and structured relationship between educational and healthcare provider institutions. This model is done to reduce the theoretical-practical gap in nursing. The present study aimed to explore the experiences of nursing students.

**Methods:**

In a conventional content analysis, 11 undergraduate nursing students, 7 graduate nursing students, and 4 doctoral students were selected. Data was collected through focus group discussions and individual interviews.

**Results:**

The findings from analyzing the students’ experiences who had received education using this model resulted in four main categories: the feeling of being a nurse, an integrated and collaborative clinical education platform, the development of nursing clinical education, and educational challenges.

**Conclusion:**

The TPSN model provides a suitable platform for nursing clinical education. This model helps students integrate theoretical knowledge with clinical practice and helps them act as professional nurses in the future.

**Supplementary Information:**

The online version contains supplementary material available at 10.1186/s12912-024-01810-6.

## Background

Nursing education has two components: theoretical education and clinical education. The theoretical knowledge that nurses learn creates the foundation of their proper practices, and their proper practice creates the foundation of efficient nursing [1], which requires the proper integration of theoretical knowledge into practice [2].

The inefficiency of teaching-learning methods and learning experiences makes it difficult for nursing students and nurses to integrate learned theoretical knowledge into clinical practices. This problem in the nursing profession is considered the theory-practice gap [3]. This gap adversely affects the education of competent nurses [4]. Studies in Iran also show the theory-practice gap in nursing [5]. Lack of effective interaction between care institutions and colleges has been reported as the main cause of this gap [6].

A review of the literature shows that, over the years, various approaches, such as collaborative clinical teaching and academic-practice partnership patterns, have been proposed in nursing education to reduce this gap [7]. Collaborative clinical teaching is a model in which specific clinical nurses are involved in students’ clinical teaching [8]. Some of the strategies for this model include preceptorship, internship, and the clinical teaching associate (CTA) method [9]. In the academic-practice partnership model, there is a two-way interaction between healthcare and academic institutions [10]. It included strategies such as joint appointments, the clinical scholarship model, and the dedicated education unit [11]. Despite the positive effects of these approaches, studies have shown that this gap still needs to be addressed in nursing education worldwide [12, 13].

In Iran, in response to the theory-practice gap in nursing, Recruitment regulation Clinical Faculty Members was designed and notified by the Ministry of Health and Medical Education [14]. This regulation had some problems, such as the vagueness of the clinical faculty member’s role, the obscurity of the interaction and participation of academic and healthcare settings, and a lack of clear strategies for implementing the regulation and achieving the goals [15].

To develop the role of faculty members and reduce the theory-practice gap in nursing, a practical model was designed and implemented within the framework of a participatory paradigm and an action research (PARENT study) between a nursing school and a hospital. The TPSN (Teacher, Patient, Student, Nurse) model is an integrated academic-practice model, a dynamic and interactive model for accountability in the nursing discipline [16]. Previous studies have shown that the use of the TPSN model in an integrated system will improve clinical education and the quality of nursing care, provided that the role and position of nursing instructors in clinical fields are clearly defined, the official scope of their powers and responsibilities is determined, and mentoring and preceptorship programs are regularly implemented [17].

The TPSN model has three components: mentoring, preceptorship, and integrated clinical education. Clinical education of undergraduate nursing students, as one of the main components of this model, is included within the components of preceptorship and integrated clinical education [18].

Particular strategies such as cascade education, journal clubs, nursing grand rounds, and education based on the nursing process are used for the nursing students’ integrated clinical education [16]. The attending nurse is central to educating undergraduate, graduate, and doctoral students. The attending nurse’s educational activities are used to empower all students. Masters and doctoral students participate in education with specific duties. As attending nurse’s assistants, doctoral students provide graduate and undergraduate students with journal clubs, grand rounds, and practical education procedures. The master students act as role models for undergraduate students by guiding them, fully implementing the nursing process, and helping them to implement practical procedures. The education of undergraduate students follows the nursing process, which is transferred to them through cascade education [18].

The preceptorship component is formed through the interaction of nurses and other health service providers with students and patients. Its structure is based on the modified preceptorship model, and it considers clinical nurses as role models to educate nursing students. Nursing services are based on the nursing process, and nurses are role models who implement the nursing process for students and teach them practical procedures [17].

The innovative model of TPSN is presented to train conversant, capable, and competent nurses who can integrate theoretical and practical knowledge and provide quality and safe nursing care [17]. It is necessary to identify how this model affects educational components and understand its strengths and weaknesses for its more effective use. Nursing students who have been educated using this model constitute the primary information sources. Discovering the facts from students’ experiences and using them to improve the model can provide a more efficient educational model. Therefore, qualitative approaches, such as conventional content analysis, are comprehensive and complete to get a clear and accurate picture of the truth. The purpose of this study was to explore the experiences of nursing students about clinical education based on the TPSN model.

## Methods

### Design of the study

As part of a national project (in the form of a nursing doctoral thesis) that has been carried out with the methodology of practical or collaborative action research (parent study), this study was conducted in 2020 at Tabriz University of Medical Sciences with the ethics code of IR.TBZMED.REC.1397.643.

The present study has an inductive qualitative design. To be able to illuminate the nursing students’ experiences of education based on the TPSN model, focus groups and individual interviews were used as data collection methods. Based on the study’s purpose, conventional content analysis was employed to explore the nursing students’ experiences. When there is no established theory or research literature on a particular topic, content analysis can be used to infer or deduce from qualitative or quantitative data related to that topic. Qualitative content analysis is a systematic approach to analyzing qualitative data. It can be used to analyze descriptive and explicit content, classes, latent content, interpretively produced content, and provide production themes [19]. In addition, a purposeful sample was selected from students who had completed the educational course according to the TPSN model. The study was reported according to the Consolidated Criteria for Reporting Qualitative Research checklist [20] (Supplementary Material 1).

### Study settings and participants

The project is carried out in the cardiology ward of Shahid Madani Hospital, which is affiliated with Tabriz University of Medical Sciences. A purposeful sample was selected from students who had taken the educational course according to the TPSN model. We used a purposeful sampling strategy to ensure information richness, i.e., by recruiting participants with education level, experience, and interest concerning the study aim. Inclusion criteria included a willingness to voluntarily participate in the study and having at least one month of education, according to the TPSN model. One of the authors (MN) telephoned the students to ask if they were willing to participate in the interviews. Students who were interested in participating were asked to sign and return the consent form. The sample included 11 undergraduate nursing students, 7 master’s students, and 4 doctoral students in nursing. Demographic characteristics and meeting types are shown in Table 1.

**Table 1:** Characteristics of students participating in the study and meeting types

	No. of employees	No. of sessions	Education level		
			Bachelors	Masters	PhD
Focus group session (group 1)	6	2	3	2	1
Focus group session (group 2)	6	2	3	2	1
Focus group session (group 3)	7	2	4	2	1
Individual interview	1	1	*		
Individual interview	1	1		*	
Individual interview	1	1			*

### Data collection

Focus group discussions were chosen for data collection. We considered focus groups as a suitable method to explore the experiences of nursing students in clinical education based on the TPSN model. Including participants from various education levels allowed the participants to exchange thoughts and ideas, thus extending the understanding of the research question [21], and it is used as a qualitative approach to gain a deep understanding of social issues and appropriate information about students’ experiences and perspectives [22]. Three groups were selected for focus group discussions, which continued until data saturation was reached. Two sessions, each of which lasted 90 min, were held for each group, and the interviews were carried out in a classroom in the faculty. Focus groups included two researchers as a moderator and a co-moderator. The moderator was engaged in guiding the group discussion, encouraging the students to participate in the discussions, stimulating discussions in the group, facilitating communication, creating interaction, and keeping the group dynamic. The co-moderator took notes and provided comments to guide and focus the discussions on the topic. After the fourth focus group, the data was regarded as saturated. To confirm that no further information would appear, the fifth and sixth interviews were conducted. Individual interviews were also conducted to provide a more private environment for students to express their experiences. A bachelor’s student, a master’s student, and a doctoral student participated in individual interviews to collect the required information. Each interview lasted 45 min, and the interviews were carried out in a classroom in the faculty. The resulting information did not add a new category. All the interviews and discussions were recorded and transcribed by the researchers. Transcribed files were checked for accuracy against recordings and then anonymized.

The focus groups and individual interviews were conducted using an in-depth and semi-structured interview guide. The interview guide was developed based on a previously conducted literature review, identified knowledge gaps, and expert opinions. To ensure the quality of the interview guide, the first focus group and individual interview served as a test interview, but as no subsequent changes were made, it has been included in the study process. The focus groups and individual interviews started with open-ended questions and were followed by more detailed ones. Interviews included broad questions such as “Please describe your experiences with clinical education according to the TPSN model?”, “What are the most important challenges you faced during your clinical education?”, “What causes these challenges mainly?” and “Explain more?”

Before conducting the focus groups and interviews, the students were given basic information about the study, such as the study’s purpose and methodology. In addition, they were assured of the confidentiality of their information, including the recorded voices, and informed that they were free to participate or leave the study. The study followed accepted ethical standards, as outlined in the Declaration of Helsinki. The purpose of the study was explained to the participants, and written informed consent was obtained.

### Data analysis

This study uses conventional content analysis to analyze the data. The qualitative analysis was carried out in several steps, inspired by the methodological structure provided by Graneheim and Lundman [23]. At the end of each session, the recorded interviews were transcribed verbatim and then analyzed. The researcher reviewed and examined the transcribed texts several times to achieve a complete understanding of them through an inductive approach (this step was carried out by MN, FB, and AP individually, and then all authors came together to discuss the findings). Then, the meaning units (e.g., words, sentences, or paragraphs) relating to the questions were identified and extracted (this step was carried out by MN, FB, and AP individually, and then all authors came together to discuss the findings). In the next step, the meaning units were condensed while preserving the core (this step was carried out by AP individually, and then all authors came together to discuss the findings). Condensed meaning units were abstracted into codes and categories. Comparable codes representing similar concepts were divided into subcategories, which were then combined to form a category (all the authors discussed). The MAXQD software version 10 software was used to manage the data.

### Reliability and validity

This study uses the scientific accuracy criteria in the qualitative parts of the research, as presented by Lincoln and Guba [24]. To ensure reliability, four requirements of creditability, dependency, conformability, and transferability are required, according to this suggestion.

The credibility of the findings was supported by including direct quotations from the transcribed text. Also, the researchers confirmed the data’s credibility through long-term contact with the participants and gaining their trust. Peer checks were used to confirm the data’s dependability. The study team conducted regular peer reviews to ensure that newly discovered data was discussed thoroughly. Biweekly peer checks were conducted to ensure that the study team had a full discussion of the newly discovered data. The background information and the researchers’ interests in relevant subjects were used to confirm the data’s conformability. The opinions of the team’s experts in the field of qualitative research were used to continuously review and compare the similarities and differences of the data and classes. The researchers described the research environment, the participants’ characteristics, and the collection and analysis processes to increase the transferability of the results.

## Results

The results of the information analysis divided the nursing students’ experiences in clinical education according to the TPSN model into four categories: feeling of being a nurse, integrated and collaborative clinical education platform, development of nursing clinical education, and educational challenges.

Students’ experiences of clinical education using the TPSN model revealed a fundamental change in clinical education. Table 2 shows the categories and subcategories resulting from the analysis of students’ experiences.

**Table 2:** Categories and subcategories resulting from the analysis of students’ educational experiences using the TPSN model

Feeling of being a nurse	Confidence in performing tasks
	Feeling of professionalism
	Feeling of being empowered to make a change
	Positive attitude toward becoming a nurse
An integrated and collaborative clinical education platform	Constructive interaction
	Supportive atmosphere
	Mutual cooperation between the faculty and the hospital
Development of nursing clinical education	The enhanced education of practical skills
	integrating theoretical knowledge in practice
	Promoting critical thinking
	Improving practical skill learning with cascade education
	Implementing nursing process in practice
Educational challenges	Inadequate ability of nurses to implement the nursing process
	Inadequacy of technology for recording
	Role interference

### Feeling of being a nurse

This category shows that the implementation of the TPSN model provides a suitable platform for the identification of students to accept nursing roles during clinical education.

#### Confidence in performing tasks

The students found that the strategies included in the model components (i.e., mentorship, preceptorship, and integrated clinical education) prepared them to perform nursing tasks. Undergraduate students said that they were supported by professors, masters and doctoral students, and clinical nurses and considered them as their scientific and practical role models. The students stated that the education they received from skilled people before doing the clinical task and their guidance during the task increased their self-confidence in nursing. Master and doctoral students also stated that the nursing professor was a suitable role model for them in teaching and increased their self-confidence in teaching undergraduate students.

Two students described the situation as follows:

“Before, when I was given a clinical task, I saw myself alone and was afraid to make a mistake, even though I knew how to do that work. However, now, I already know I can do the task, and there is someone to check my work with him/her, and I know that the professor, master and doctoral students, and nurses monitor my work and prevent me from making mistakes.” (B4).

The professor’s behavior in education was a role model for me, and I quickly learned how to teach the students. In addition, I trust myself as an educator in nursing. (D32)

#### Feeling of professionalism

The students could influence the patient’s health by performing the clinical tasks that were assigned to them. On the other hand, they considered themselves responsible for providing proper care to patients. They see themselves as part of the care system in the ward. In addition, they were respected by the ward care system and patients like a nurse. In this regard, a student said:

“When helping the nurses to take care of the patient, they respected me because they knew that my professor had given me the necessary education and that the master’s and doctoral students were supporting me.” (B7).

#### Feeling of being empowered to make a change

Clinical education using this model helped the students identify their weaknesses during the clinical task. In addition, the availability of professors, masters, doctoral students, and clinical nurses provided them with role models they could use as their scientific reference and make necessary changes in their skills.

On the other hand, students with the necessary skills believed they could be effective and more active in caring for patients. A student described the situation as follows:

“When the teaching of the nursing process was completed, I performed the nursing process better than before for the patient entrusted to me, and I know I have effectively cared for my patients.” (M27).

#### Positive attitude toward becoming a nurse

The students considered their nursing work a task with a positive return and believed that nursing is valuable work for the patient. They had a positive attitude toward the importance of the nurses’ role in the patient’s recovery.

The importance and status of nursing enhanced students’ perspectives, as they saw the power of nursing professors and nurses and their effectiveness in managing the patient’s condition. In this regard, two students stated that:

“When I became a nursing student, I thought nurses only carried out the doctor’s orders for the patient. However, now, I know everyone works together for the care and treatment of the patient and that nurses have an essential role in the health system.” (M23).

“Everyone in the clinical ward respected nursing professors, whose role was not only to teach us but he/she was our scientific reference with high power.” (D31).

### An integrated and collaborative clinical education platform

This category shows that implementing the TPSN model increased the coordination between the nursing faculty’s teaching team, the hospital health system, and the nursing team. Creating the necessary interaction between different parts provides the students’ clinical education with proper support from the healthcare team. The nursing schools and healthcare teams cooperated to educate in an integrated and collaborative manner.

#### Constructive interaction

The education and healthcare teams cooperated and designed the patient care plan through the nursing process. The plan provided a common language between individuals. The nurses’ cooperation in designing and implementing this plan eliminated the communication gap between the education and healthcare teams. On the other hand, implementing programs such as journal clubs, grand rounds, morning rounds, bedside rounds, and bedside clinical rounds with the presence of the nursing professor created constructive interactions between students and nurses.

In this regard, a student said: “At the beginning of the internship, when I was assigned a patient, I and the patient’s nurse planned together about the care plan and nursing process for the patient. In the morning round, the nursing professor supervised my work, and I cooperated with the nurse until the end of the internship. If I had any problems, I would get help from a master’s or PhD student.” (B11).

#### Supportive atmosphere

The placement of nurses in the educational team increased their sense of belonging to the education team of students. As a result, the ward’s atmosphere was educational, which increased students’ adaptability to the environment. The nursing system considered itself a part of student education, which increased their education support.

In this regard, a student said: “Nurses were like professors during my internship and taught me how to do clinical work, and I could ask them my questions. When my professor was unavailable, I could easily turn to them, and they would help me.” (B8).

#### Mutual cooperation between the faculty and the hospital

The effective cooperation between the college’s educational team and the hospital provided a structured relationship for providing two-way services. This structured relationship between the college’s educational and nursing teams created a two-way teaching-learning process.

The faculty’s education team considered itself responsible for patient care. On the other hand, the treatment team in the hospital, as part of the clinical education system, provided appropriate education to the students while providing services to the patients.

In this regard, another student said: “Nurses treated me like a member of their group, while I used to be their burden. The more we helped them provide services, the more they taught us.” (B2).

### Development of nursing clinical education

The hardware and software changes in nursing clinical education using the TPSN model made the educational efforts and activities more coordinated. Education through the TPSN model improved students’ learning, practical skills, and growth.

#### The enhanced education of practical skills

Educating nursing students on clinical work and practical skills by experienced nurses had the advantage that students could learn clinical work from people with more expertise, which increased their opportunity to do clinical work.

In this regard, a student said: “The nurses taught us all the clinical work they carried out for the patient. After that, most of the clinical work was entrusted to us, and the nurses always helped us. Now, I feel that I have learned many practical skills and can work independently.” (B9).

#### Integrating theoretical knowledge in practice

The students believed that converting theoretical knowledge into performing clinical tasks is facilitated using the strategies existing in the TPSN model. Using theoretical science in nursing care, such as nurses’ activities in the preceptorship position, has been realized through providing objective context.

In this regard, a student said: “At first, the nature of theoretical science and clinical practice was different for me, but now I know that I have to use my theoretical knowledge to care for patients. For example, when I choose an appropriate nursing diagnosis for a patient, I also use the appropriate intervention for him.” (B5).

#### Promoting critical thinking

Many students stated that their ability to manage new situations increased during their internship. They could assess patients, obtain appropriate evidence for their problems, and apply effective solutions. Using the nursing process during care provision would increase students’ abilities and foster their critical thinking in clinical care.

In this regard, a student said: “When I was caring for a patient entrusted to me, the patient suddenly suffered from shortness of breath. It informed the nurse in charge of the patient about his emergency condition. I reviewed and performed everything I had to do and checked it with the nursing professor and the relevant nurse. My plan and the nurse’s plan for the patient were the same, so we started caring together.” (B9).

#### Improving practical skill learning with cascade education

Access to graduate and doctoral students would increase students’ learning resources. The students stated that they referred to graduate and doctoral students to understand the nursing professor’s teaching better. It was easier for students to communicate and convey their needs to undergraduate students as they were more accessible.

In this regard, a student said: “I would prefer to ask my questions of the doctoral and master students. There are many undergraduate students, and the nursing professor is not always available to ask our small questions, but we can easily ask questions from graduate and doctoral students. They were always teaching us.”

#### Implementing the nursing process in practice

The nursing process in the TPSN model was a common language between individuals to provide nursing care to patients. The students’ observations helped them learn how to apply their knowledge in the nursing process. The nursing process was the basis for education, and clinical education was conducted for students accordingly.

In this regard, a student said: “Before, I knew only how to write a nursing diagnosis based on the nursing process, but now I know how to implement a nursing process completely for my patients.” (M26).

### Educational challenges

#### Inadequate ability of nurses to implement the nursing process

The nursing process is the basis of nursing care and clinical education in the TPSN model. The ward’s nurses used to not implement the nursing process completely before implementing this model, due to their inability. Although the nurses were given the necessary education in advance, students stated that, in some cases, they wrongly performed parts of the nursing process.

In this regard, a student said: “Sometimes, when I wrote the nursing process with the relevant nurses, I realized that they mostly use repetitive diagnoses like “anxiety due to unfamiliarity with the environment.” (M26).

#### Inadequacy of technology for recording

Taking care of the patient was based on the nursing process and was recorded accurately. This model required writing the records, which slowed the process and increased the workload by increasing the need to write.

In this regard, a student said: “There was so much paperwork that prevented me from considering the plan and taking care of a patient hospitalized for a long time. Nurses had to allocate more time to write the records.” (B9).

#### Role interference

Students stated that they sometimes got confused, as many people (e.g., professors, doctoral and master students, and nurses) could ask their questions or deliver their assignments.

In this regard, a student said: “When I asked a question from a nurse, she said I should ask my professor.” (B10).

## Discussion

This study explains the nursing students’ experiences during clinical education using the TPSN model. This model is a new and innovative model to reduce the theory-practice gap in nursing and provides a framework for the development of clinical curricula for nursing students.

The findings from analyzing the students’ experiences who had received education using this model resulted in 4 main categories: feeling of being a nurse, integrated and collaborative clinical education platform, development of nursing clinical education, and educational challenges.

Clinical education is central to nursing education, which aims for students to acquire and develop professional skills to provide appropriate nursing care [25]. Training appropriate nursing forces to meet the health care system’s needs requires empowering students in different dimensions, such as combining nursing science with practice, clinical reasoning, critical thinking, ethical practice, and evidence-based practice [9, 26]. Nursing schools must provide suitable platforms to acquire these capabilities. Various studies show that nursing education is faced with the challenge of the gap between theory and practice, and due to this, these abilities are not fully manifested in students [14]. Deborah Spence et al. showed that suitable clinical education models are necessary to overcome nursing education’s challenges [27]. The experiences of students who participated in the study show that education based on the TPSN model provides a suitable platform to use the nursing process for providing care, and the students could apply their theoretical knowledge learned in practice through critical thinking. The nurses’ participation in education increased their opportunity to do practical work for students, and cascade education improved their access to practical references in practical work.

In addition to being influenced by the educational activities of the nursing instructor, effective clinical education in nursing is also influenced by the clinical educational environment. The cooperation and coordination of the clinical environment with educational activities facilitate realizing educational goals [28]. Despite the importance of this issue, the existing gap between nursing schools and clinical environments is an important concern that challenges nursing education [10]. The study results show that integrating education and the provision of nursing services increases nurses’ participation and cooperation level in education, providing nursing education on an integrated platform where the faculty and hospital are present.

The students found that education using the TPSN model increased their self-confidence in performing nursing tasks and creating changes. The characteristics they see growing in them brought them closer to nursing professionalism and provided them with a suitable perspective for becoming a nurse in the future. Various studies have shown that it is essential for students to adapt to the nursing role to become efficient nurses, which is affected by various factors [29, 30]. Lee et al. showed that students’ self-confidence is essential in transforming them into nurses, with education being one of the main effective factors in this process [31]. The results of this study showed that this model provides a suitable basis to increase students’ self-confidence to make changes. On the other hand, many characteristics, such as responsibility, commitment, ability, and attitude toward nursing, are essential in acquiring a professional identity [32]. Students’ experiences show that education using this model empowers students to become professionals during their education.

The students’ experiences revealed that the TPSN model, besides providing a suitable platform for clinical education in nursing, has faced some challenges due to its new nature. The nursing process is the key to providing care in this model. Nurses’ lack of full use of this model before its implementation makes it challenging for them. The results of a study related to parent study showed that educating nurses and the continuous use of education increase their ability to implement the nursing process over time [17]. On the other hand, the paper registration system made the implementation process time-consuming and challenging. Computer-based registration facilitates the implementation of the nursing process, increases its accuracy, and reduces time waste [33]. Another challenge raised in this study was the interference of individuals’ roles in education. The simultaneous presence of attending professors, masters and doctoral students, and a nurse increased the overlapping of education tasks. Although students were taken role tasks at the beginning of the study, these tasks should be re-examined by the research team, and their overlapping should be reduced.

## Conclusion

The results showed that internship in nursing is an essential course in educating individuals as nurses, and the TPSN model provides a suitable platform for this education. The use of appropriate models in education makes teaching-learning methods regular and makes education more efficient. The TPSN model revolutionized the process of clinical nursing education by creating a collaborative platform between the nursing school and the hospital. This model decreases nursing challenges by reducing the theory-practice gap. Therefore, this study’s results will help provide macro-policies for nursing education. Providing clinical education using this model contributes to training nursing forces with the necessary qualifications. As a result, the resulting changes will be reflected in the care outcomes the health system provides for society.

### Electronic supplementary material

Below is the link to the electronic supplementary material.

Supplementary Material 1

## Data Availability

The datasets used and/or analyzed in the current study are available from the corresponding author upon reasonable request.

## References

[1] Saifan A, Devadas B, Daradkeh F, Abdel-Fattah H, Aljabery M, Michael LM (2021). Solutions to bridge the theory-practice gap in nursing education in the UAE: a qualitative study. BMC Med Educ.

[2] Hashemiparast M, Negarandeh R, Theofanidis D (2019). Exploring the barriers of utilizing theoretical knowledge in clinical settings: a qualitative study. Int J Nurs Sci.

[3] Eraut M. Transfer of knowledge between education and workplace settings. Knowledge, values and educational policy. edn.: Routledge; 2012. pp. 65–84.

[4] Greenway K, Butt G, Walthall H (2019). What is a theory-practice gap? An exploration of the concept. Nurse Educ Pract.

[5] Safazadeh S, Irajpour A, Alimohammadi N, Haghani F (2018). Bridging the theory-practice gap in Iranian emergency nursing education. ARYA Atherosclerosis.

[6] Khodaei A, Mansourain M, Ganjei S, Asgari H (2016). Strategies for decreasing gap between theory & clinical performance from the viewpoints of nursing students in tabriz university of medical sciences. Res Med Educ.

[7] Saifan A, Devadas B, Daradkeh F, Abdel-Fattah H, Aljabery M, Michael LM (2021). Solutions to bridge the theory-practice gap in nursing education in the UAE: a qualitative study. BMC Med Educ.

[8] Rich ME, Lamiman K (2019). Five concepts for collaborative clinical teaching. Clin Teach.

[9] Oermann MH, Shellenbarger T. Clinical education in nursing: current practices and trends. Clin Educ Health Professions: Theory Pract 2020:1–20.

[10] Gursoy E (2020). Partnership between academic nursing and clinical practice: a qualitative study. JPMA J Pakistan Med Association.

[11] Pedregosa S, Fabrellas N, Risco E, Pereira M, Dmoch-Gajzlerska E, Şenuzun F, Martin S, Zabalegui A (2020). Effective academic-practice partnership models in nursing students’ clinical placement: a systematic literature review. Nurse Educ Today.

[12] Grant M, Leigh J, Murray C, Howarth M (2007). The role of the academic in clinical practice: a systematic review. Medicine.

[13] Lakdizaji S, Sh G, Ghojazadeh M, Parchebafieh S (2009). Nursing students’ satisfaction with clinical teaching associated model. Nurs Midwifery J.

[14] Alimohammadi N, Khorasani P, Irajpour A, Hashemi MS, Yazdannik A, Afshari A (2022). Factors affecting the implementation of clinical nursing faculty: a step to improve the quality of clinical education. Iran J Nurs Res.

[15] Tajabadi A, Vanaki Z, Aghaei MH, Roshanzadeh M (2018). Clinical faculty member, as a challenging opportunity in nursing education: designing a perspective. Iran J Nurs Res.

[16] Zamanzadeh VVL, Ghahramanian A, Namadi Vosoughi M, Hazrati M, Shabanloui R et al. Presenting and implementing a model for integrating theoretical and clinical education in nursing. In: *Responsive Medical Education Conference*: 2018; Tabriz: Tabriz University of Medical Science; 2018.

[17] Namadi-Vosoughi M, Zamanzadeh V, Valizadeh L, Lotfi M, Ghahramanian A, Pourmollamirza A, Taleghani F, Bagheriyeh F, Avazeh M. The impact of institutionalizing the nursing process based on TPSN model on the quality and quantity of nursing diagnoses. Nurs Open 2023. 10.1002/nop2.1796 PMC10333878 37170427

[18] Vosoughi MN, Zamanzadeh V, Valizadeh L, Ghahramanian A, Lotfi M, Bagheriyeh F, Pourmollamirza A (2022). An introduction to the TPSN model: a comprehensive approach to reducing the theory-practice gap in nursing. BMC Nurs.

[19] Lindgren B-M, Lundman B, Graneheim UH (2020). Abstraction and interpretation during the qualitative content analysis process. Int J Nurs Stud.

[20] Tong A, Sainsbury P, Craig J (2007). Consolidated criteria for reporting qualitative research (COREQ): a 32-item checklist for interviews and focus groups. Int J Qual Health care: J Int Soc Qual Health Care.

[21] Krueger RA, Casey MA. Focus groups: a practical guide for Applied Research. SAGE; 2014.

[22] Nyumba O, Wilson T, Derrick K, Mukherjee CJ (2018). The use of focus group discussion methodology: insights from two decades of application in conservation. Methods Ecol Evol.

[23] Graneheim UH, Lundman B (2004). Qualitative content analysis in nursing research: concepts, procedures and measures to achieve trustworthiness. Nurse Educ Today.

[24] Schwandt TA, Lincoln YS, Guba EG. Judging interpretations: but is it rigorous? Trustworthiness and authenticity in naturalistic evaluation. New Dir Evaluation. 2007;2007(114):11–25.

[25] Shadadi H, Sheyback M, Balouchi A, Shoorvazi M (2018). The barriers of clinical education in nursing: a systematic review. Biomed Res.

[26] Mann CM, Sullivan SS (2021). Promoting evidence-based practice in clinical education at a hospice designated education unit. J Hospice Palliat Nurs.

[27] Spence D, Zambas S, Mannix J, Jackson D, Neville S (2019). Challenges to the provision of clinical education in nursing. Contemp Nurse.

[28] Kamariannaki D, Alikari V, Sachlas A, Stathoulis J, Fradelos E, Zyga S. Motivations for the participation of nurses in continuing nursing education programs. Archives Hellenic Medicine/Arheia Ellenikes Iatrikes 2017, 34(2).

[29] Hampton KB, Smeltzer SC, Ross JG (2021). The transition from nursing student to practicing nurse: an integrative review of transition to practice programs. Nurse Educ Pract.

[30] Urban RW, Barnes DM (2020). Transition to practice: the lived experience of new graduate nurses in early solo flight. J Nurses Prof Dev.

[31] Lee T, Lee SJ, Yoon YS, Ji H, Yoon S, Lee S, Ji Y (2023). Personal factors and clinical learning environment as predictors of nursing students’ readiness for practice: a structural equation modeling analysis. Asian Nurs Res.

[32] Fitzgerald A. Professional identity: A concept analysis. In: *Nursing forum: 2020*; 2020: 447–472. 10.1111/nuf.12450 32249453

[33] Lopez M, Jimenez J-M, Fernández-Castro M, Martin-Gil B, Garcia S, Cao M-J, Frutos-Martin M, Castro M-J (2020). Impact of nursing methodology Training Sessions on Completion of the Virginia Henderson Assessment Record. Nurs Rep.

