# Supplementary material for: Nursing students’ experiences from clinical education using the TPSN model
Source: BMC Nurs. 2024 Mar 4;23:155. doi: 10.1186/s12912-024-01810-6 (PMC10910728; doi:10.1186/s12912-024-01810-6)
Supplement: Supplementary file 1 — Supplementary Material 1 [file 12912_2024_1810_MOESM1_ESM.docx]

**Supplementary Material**

COREQ checklist

| **No.** | **Item** | **Description** | **Described in text (section)** |
| --- | --- | --- | --- |
| **Domain 1**: Research team and reflexivity | | | |
| 1 | Interviewer | MN, FB | Methods, Structure of the focus group interviews and individual interviews: p.6 |
| 2 | Researchers’ credentials (involved in data analysis) | VZ: Professor Of Nursing,  LV: Professor Of Nursing,  AGh: Associate Professor Of Nursing,  MN: PhD candiadate in nursing,  AP: PhD candiadate in nursing,  FB: Assistant Professor Of Nursing | Methods,  Data analysis  P. 7-8 |
| 3 | Researchers’ occupation (involved in data analysis) | VZ: Professor Of Nursing,  LV: Professor Of Nursing,  AGh: Associate Professor Of Nursing,  MN: PhD candiadate in nursing,  AP: PhD candiadate in nursing,  FB: Assistant Professor Of Nursing | Methods,  Data analysis  P. 7-8 |
| 4 | Gender | VZ: Male,  LV: Female,  AGh: Female,  MN: Female,  AP: Female,  FB: Female | No |
| b5 | Experience and training | All author specialized in education in nursing and expertise in qualitative data analysis | No |
| 6 | Relationship established | No previous relationship established with the participants | No |
| 7 | Participants’ knowledge of the interviewer | Participants knew the professional background  of the interviewer | No |
| 8 | Interviewer characteristics | MN and FB conduct research on education in nursing | No |
| **Domain 2**: Study design | | | |
| 9 | Methodological  information and theory | See body of text | Methods: p. 5  Data analysis: p. 7-8 |
| 10 | Sampling | See body of text | Methods, Study settings and participants  : p.5-6 |
| 11 | Method of approach | See body of text | Methods,  Data analysis: p.7-8 |
| 12 | Sample size | See body of text, Table 1 | Methods, Study settings and participants: p. 5-6, table 1 |
| 13 | Non-participation | None refused to participate or dropped out | No |
| 14 | Setting of data collection | See body of text | Methods, Study settings and participants: p.5-6  Data collection:p.6-7 |
| 15 | Presence of non-participants | Not applicable |  |
| 16 | Description of sample | See body of text, Table 1 | Methods, Study settings and participants: p.5-6, table 1 |
| 17 | Interview guide | See body of text | Methods, Data collection: p.6-7 |
| 18 | Repeat interviews | No repeat interviews | No |
| 19 | Audio/visual recording | See body of text | Methods, Data collection:p.6-7 |
| 20 | Field notes | No | No |
| 21 | Duration | See body of text | Methods, Data collection:p.6-7 |
| 22 | Data saturation | See body of text | Methods, Data collection:p.6-7 |
| 23 | Transcripts returned | Not performed | NO |
| **Domain 3**: Analysis and findings | | | |
| 24 | Number of data coders | See body of text | Methods,  Data analysis: p.7-8 |
| 25 | Description of coding tree | See body of text and Table 2 | Methods,  Data analysis: p.7-8 ,Table 2 |
| 26 | Derivation of themes | See body of text | Methods,  Data analysis: p. 7-8, Table 2 |
| 27 | Software | MAXQDA | Methods,  Data analysis: p.7-8 |
| 28 | Participant checking | Not performed | NO |
| 29 | Quotations presented | See body of text | Results: p. 8-15 |
| 30 | Consistency between  data and findings | See body of text | Results: p. 8-15 |
| 31 | Clarity of major themes | See body of text, Table 2 | Results: p. 8-15, Table 2 |
| 32 | Clarity of minor themes | See body of text  . | Results: p. 8-15 |
